# The prevalence of *Staphylococcus aureus* and the emergence of livestock-associated MRSA CC398 in pig production in eastern China

**DOI:** 10.3389/fmicb.2023.1267885

**Published:** 2023-12-15

**Authors:** Lina Zheng, Zhongyi Jiang, Zhenyu Wang, Yang Li, Xinan Jiao, Qiuchun Li, Yuanyue Tang

**Affiliations:** ^1^College of Bioscience and Biotechnology, Yangzhou University, Yangzhou, Jiangsu, China; ^2^Jiangsu Key Laboratory of Zoonosis/Jiangsu Co-Innovation Center for Prevention and Control of Important Animal Infectious Diseases and Zoonoses, Yangzhou University, Yangzhou, Jiangsu, China; ^3^Key Laboratory of Prevention and Control of Biological Hazard Factors (Animal Origin) for Agri-food Safety and Quality, Ministry of Agriculture of China, Yangzhou University, Yangzhou, Jiangsu, China; ^4^Joint International Research Laboratory of Agriculture and Agri-Product Safety, Yangzhou University, Yangzhou, Jiangsu, China

**Keywords:** *Staphylococcus aureus*, LA-MRSA, pig production, clonal complex 398, antibiotic resistance

## Abstract

Livestock-associated *Staphylococcus aureus* (LA-MRSA) has been of increasing concern due to its potential risk to humans. This study investigated the prevalence of MRSA in pig production in Eastern China and determined the genomic characteristics of pig-associated MRSA isolates by whole-genome sequencing (WGS). A total of 1,318 samples were collected from pig farms and pig slaughterhouses, and 150 *S. aureus* were identified, including 63 MRSA isolates and 87 MSSA isolates. MRSA was detected in all pig farms and pig slaughterhouses. The antimicrobial susceptibility test revealed that all MRSA isolates were multidrug-resistant. The WGS and MLST analysis demonstrated that 56 MRSA isolates belonged to clonal complex (CC) 398, and seven MRSA isolates belonged to CC9. All LA-MRSA isolates were absent of phiSa3 phage containing immune evasion cluster (IEC) and possessed an intact *hlb* gene. In addition, genes associated with Panton-Valentine leukocidin, typically indicative of human adaptation, were not detected. The analysis of antibiotic resistance genes (ARGs) demonstrated that all MRSA isolates contained multiple ARGs. All MRSA isolates had Plthe *mecA* gene and at least one tetracycline resistance gene. Both *tetM* and *tetK* were detected in all MRSA CC398 isolates, while *tetL* was detected in all MRSA CC9 isolates. The phenicol resistance gene *fexA* was detected in 51 MRSA isolates, while the linezolid resistance gene *cfr* was detected in 60 MRSA isolates. The emergence of LA-MRSA CC398 in four pig farms and one slaughterhouse in this study indicates the spread of this clonal complex in the pig production sector in Eastern China. Further investigations are required to understand the potential transmission routes of LA-MRSA CC398 within the pork production chain in China and to assess the potential risks to humans.

## 1 Introduction

Livestock-associated methicillin-resistant *Staphylococcus aureus* (LA-MRSA) is an emerging animal-associated food safety problem in many parts of the world. Particular focus has been on pork production as pigs are universally known as the main reservoir for LA-MRSA. In Europe and North America, LA-MRSA clonal complex (CC) 398 is the predominant CC type, which is associated with the primary pig industry, and is also prevalent in poultry, cattle, sheep, and animal-origin meat products (Larsen et al., 2015; Sieber et al., 2018). Meanwhile, occupational exposure during livestock production has been regarded as the critical risk factor for LA-MRSA transmission in humans and the secondary spread to the community (Aires-De-Sousa, 2017). A European study demonstrated that 21.5% of LA-MRSA from clinical isolates belonged to CC398 in the Netherlands, while the ratio was around 9.7% in Belgium and Spain, 15.6% in Slovenia, and 16.7% in Demark (Kinross et al., 2017).

In China, CC9 is the predominant LA-MRSA CC type in pig production (Zou et al., 2022). However, LA-MRSA CC398 has already been sporadically reported in several surveillance studies in different provinces in China. A prevalence study has demonstrated that 38% of pig-associated *S. aureus* isolates belonged to LA-MRSA CC398, indicating the emergence of LA-MRSA CC398 in pig production (Cui et al., 2022). Besides, LA-MRSA CC398 has also been sporadically reported in pork and bulk milk in China (Monaco et al., 2013; Van De Sande-Bruinsma et al., 2015). This study primarily aimed to investigate the prevalence of MRSA in pig farms and slaughterhouses in Eastern China based on whole-genome sequencing (WGS) analysis. *S. aureus* isolates were collected from pig farms and slaughterhouses in Eastern China and subjected to WGS and MLST analyses. Since LA-MRSA CC398 has been widely associated with the pig industry and occupational infections in humans, we investigated the phylogenetic relationship of LA-MRSA CC398 isolates from this study to human-associated MRSA CC398 and LA-MRSA CC398 from both China and other countries. The phylogenetic analysis was performed to investigate the emergence and spread of LA-CC398 in the Chinese pig industry.

## 2 Materials and methods

### 2.1 *Staphylococcus aureus* isolation and identification

Samples were collected from 2018 to 2021 at four pig farms in Jiangsu province (farms A to D) and two pig slaughterhouses in Shanghai (slaughterhouses A and B), China. A total of 1,318 samples from pig farms were collected, including 304 fecal samples, 541 anal swab samples, 105 nasal swab samples, and 113 swab samples of gloves (Table 1). A total of 255 samples from two pig slaughterhouses were collected, including 30 samples from pig carcasses before slaughtering, 167 swab samples during the pig slaughtering process, 8 swab samples of the slaughtering floor, and 50 swab samples of knives during the slaughtering process (Table 2). All swab samples were stored in collection tubes containing Cary-Blair agar. Detailed sampling information is provided in Supplementary Table S1.

**Table 1:** Prevalence of *Staphylococcus aureus* from pig farms.

Source	Sample size	No. of positive	Prevalence	MSSA	MRSA
Faeces	304	74	24.34%	50	24
Anal swabs	541	28	5.18%	0	28
Nasal swabs	105	4	3.81%	0	4
Gloves	113	5	4.42%	0	5
**Total**	**1,063**	**111**	**10.44%**	**50**	**61**

**Table 2:** Prevalence of *Staphylococcus aureus* from pig slaughterhouses.

Source	Sample size	No. of positive	Prevalence	MSSA	MRSA
Carcass	30	2	6.67%	1	1
Bleeding	10	1	10.00%	1	0
Scalding	10	1	10.00%	1	0
Depilating	30	2	6.67%	1	1
Evisceration	11	0	0.00%	0	0
Splitting	30	9	30.00%	9	0
Dressing	26	8	30.77%	8	0
Washing	20	0	0.00%	0	0
Chilling	30	7	23.33%	7	0
Floor	8	0	0.00%	0	0
Knives	50	9	18.00%	9	0
**Total**	**255**	**39**	**15.29%**	**37**	**2**

All samples were subjected to *S. aureus* isolation within 24 h, according to the literature previously described by Li et al. (2020). Briefly, each swab sample was enriched in 10 mL trypticase soy broth (TSB) with 6.5% NaCl and inoculated overnight at 37°C, 180 rpm. Ten μL enriched aliquots were inoculated on CHROMagar™ Staph aureus plates (CHROmagar, Paris, France) and incubated overnight at 37°C for the isolation of *S. aureus*. The presumptive *S. aureus* clones were identified by the presence of the *spa* gene, and MRSA was identified by the presence of the *mecA* or *mecC* gene (Tang et al., 2017).

### 2.2 *Spa* typing

All confirmed *S. aureus* isolates were analysed for *spa* typing (Supplementary Tables S2, S3). *spa* genes were detected with the primers *spa-1113f* and *spa-1514f* by the PCR program according to the previous description (Harmsen et al., 2003). The PCR product for each isolate was sequenced by Sanger sequencing (Genscript Biotech Corporation, Nanjing, China). The *spa* type was analysed by the Ridom Spa Server database (http://spaserver.ridom.de/, accessed on 4 February 2022).

### 2.3 Antimicrobial susceptibility test for MRSA

Antimicrobial susceptibility of all MRSA isolates was performed by disk diffusion according to the guidelines of the Clinical and Laboratory Standards Institute (2020). MRSA isolates from overnight TSA plates were resuspended in 0.9% saline to achieve the turbidity of 0.5 McFarland standard. The suspended cultures were evenly spread on the M.H. agar plate, and the antibiotic disk was tightly on the agar surface before being inoculated overnight at 37°C. The diameter of the antibiotic-resistant halo was measured, and results were interpreted according to the CLSI standard. A total of 10 antibiotics (Oxoid™, United Kingdom) were included: cefoxitin (FOX, 30 μg), chloramphenicol (C, 30 μg), ciprofloxacin (CIP, 5 μg), clindamycin (DA, 2 μg), erythromycin (E, 15 μg), gentamicin (C.N., 10 μg), linezolid (LZD, 10 μg), penicillin G (P, 10 units), tetracycline (T.E., 30 μg), and trimethoprim/sulfamethoxazole (SXT, 1:19, 25 μg). *S. aureus* ATCC25923 was included for quality control.

### 2.4 Whole genome sequencing of MRSA and comparative genomic analysis

All MRSA isolates were whole genome sequenced (WGS) by the Nova seq6000 (Illumina, the US). Raw reads were trimmed and filtered by the NGSQC toolkit and assembled with *de novo* assembly by SPAdes 3.15.5 (Bankevich et al., 2012). WGS data were submitted to the European Nucleotide Archive database (ENA) with the accession number PRJEB62143. The multi-locus sequence typing (MLST) was obtained by submitting the WGS data of each MRSA isolate to the database (https://pubmlst.org/saureus/, accessed on July 30, 2022). All MRSA isolates were tested for the core genome SNP analysis, and the genetic relationships of MRSA were revealed by the phylogenetic tree. To compare our MRSA CC398 isolates to the isolates from other countries, we downloaded the isolates data from Price et al. (2012). The phylogenetic tree of all MRSA isolates in this study and phylogeny was constructed by Parsnp v1.7.4 (https://harvest.readthedocs.io/en/latest/content/parsnp.html, accessed on August 3, 2022). Antimicrobial resistance genes of MRSA were detected by the ResFinder 4.0 database (Florensa et al., 2022). Virulence factors of all MRSA isolates were analysed by BLASTn against the Virulence Factors Database (VFDB) with >80% sequencing homology and coverage (Chen et al., 2005). Genes encoding virulent factors included *scn* encoding staphylococcal complement inhibitor; *chp* encoding chemotaxin inhibitory protein; *sak* encoding staphylokinase; *sea* encoding staphylococcal enterotoxin toxin A; *hlb* encoding β-hemolysin; *lukF-PV* and *lukS-PV* encoding Panton-Valentine leucocidin (PVL); tsst-1 encoding toxic shock syndrome toxin-1.

## 3 Results

### 3.1 Prevalence of *Staphylococcus aureus* in early-stage pig production

A total of 1,318 samples were collected from pig farms and pig slaughterhouses. In total, 11.38% of the samples were positive for *S. aureus*. MRSA was detected in pig samples from all farms and slaughterhouses, and no animal delivery occurred between farms and slaughterhouses (Supplementary Table S1). MSSA was detected in farm D, slaughterhouse A, and slaughterhouse B (Supplementary Table S1). In total, 63 MRSA isolates and 77 MSSA isolates were identified. The MLST analysis demonstrated that 56 MRSA isolates belonged to CC398, while 7 MRSA isolates belonged to CC9.

*S. aureus* was isolated from 10.44% of the farm samples, including 50 methicillin-susceptible *S. aureus* (MSSA) isolates and 61 MRSA isolates (Table 1). The distribution of *S. aureus* in different samples is diverse, including 24.34% from feces, 5.18% from anal swabs, 3.81% from nasal swabs, and 4.42% from gloves for workers (Table 1). At the slaughtering stage, *S. aureus* was isolated from 39 of 225 samples with a prevalence of 15.29%, including 27 MSSA isolates and 2 MRSA isolates (Table 2). Samples from splitting and dressing had the highest prevalence of *S. aureus* approximately 30%, followed by swab samples during washing, which had a prevalence of 26.67%. During the chilling step, the prevalence of *S. aureus* was 23.33% (Table 2). Other slaughtering processes with *S. aureus* positive included pig carcasses before slaughtering, bleeding, scalding, depilation, and chilling (Table 2). It should be noted that 18% of knives during the slaughtering process were *S. aureus* positive (Table 2).

### 3.2 Antibiotic resistance of MRSA isolates from pig production

Antibiotic susceptibility tests showed that all MRSA isolates were resistant to cefoxitin and penicillin. Most MRSA isolates were resistant to clindamycin (95.24%) and chloramphenicol (76.19%), while some were resistant to erythromycin (49.21%). Two MRSA isolates showed resistance to gentamicin (3.17%). One MRSA isolate YZU 4385 displayed resistance to linezolid (1.59%), while YZU4403 showed resistance to trimethoprim/sulfamethoxazole (1.59%) (Supplementary Table S4). A total of 20 multidrug-resistant (MDR) patterns were observed, with the predominant pattern of FOX-C-DA-P-TE, followed by FOX-C-DA-E-P-TE. MDR patterns of each MRSA isolate are illustrated in Supplementary Table S4.

### 3.3 Genomic characterisation of *Staphylococcus aureus* isolates from pig farms and pig slaughterhouses

**Figure 1:**
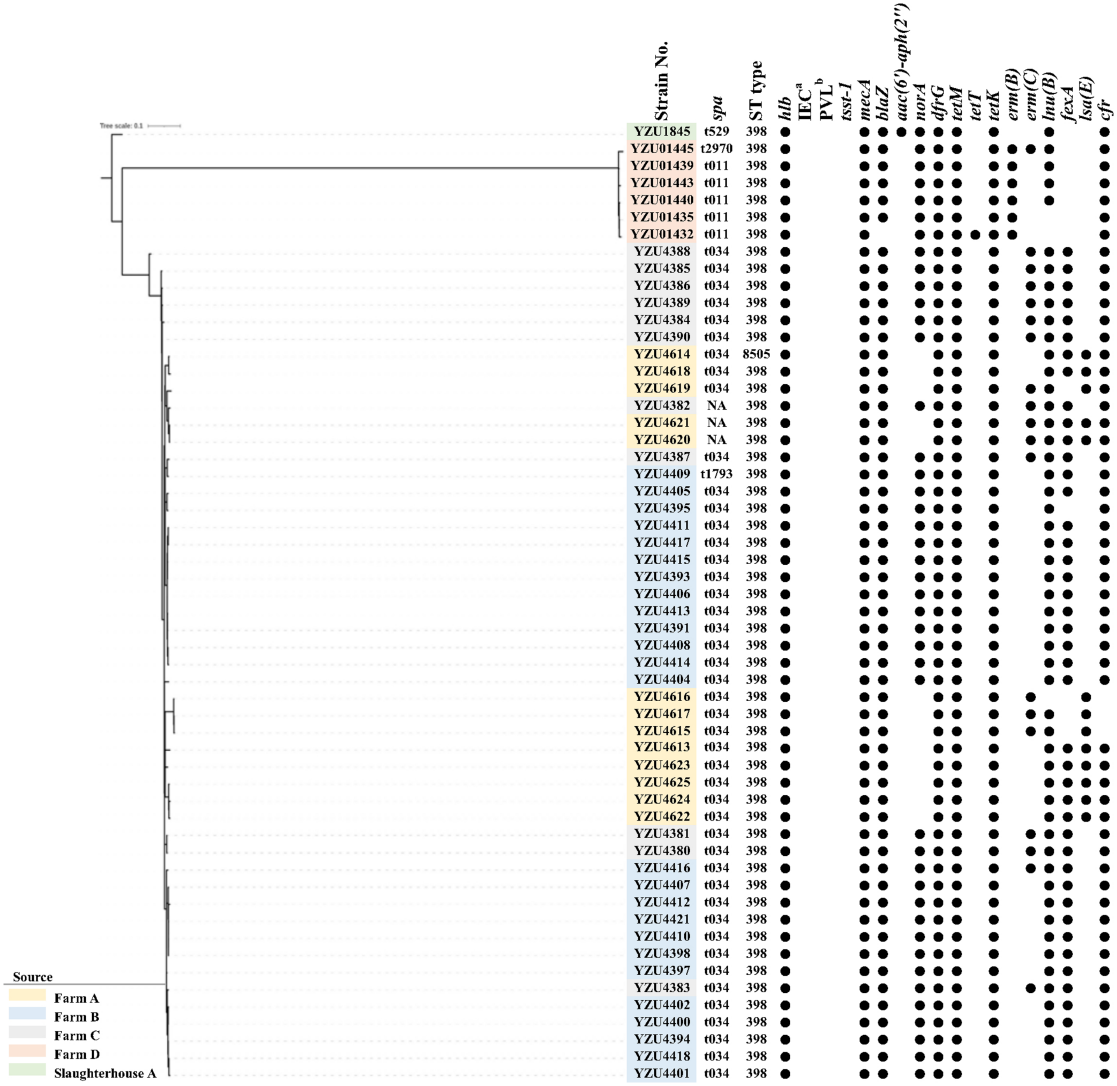
The phylogenetic tree of MRSA CC398 in terms of the core genome multi-locus sequence type (cgSNP). The filled circle (●) represents the presence of a gene. ^a^immune evasion cluster (IEC). ^b^the Panton-Valentine leucocidin (PVL).

In total, 13 *spa* types were detected in all *S. aureus* isolates (Figure 1; Supplementary Tables S2, S3). Most MRSA isolates (71.4%) belonged to *spa* type t034, followed by 11.5% belonging to t899, and 8.2% belonging to t011 (Supplementary Table S2). Eight *spa* types were detected in MSSA isolates, of which *spa* type t286 was predominant with a ratio of 32.2%, followed by *spa* type t571 (26.4%) and t899 (17.2%) (Supplementary Table S3).

**Figure 2:**
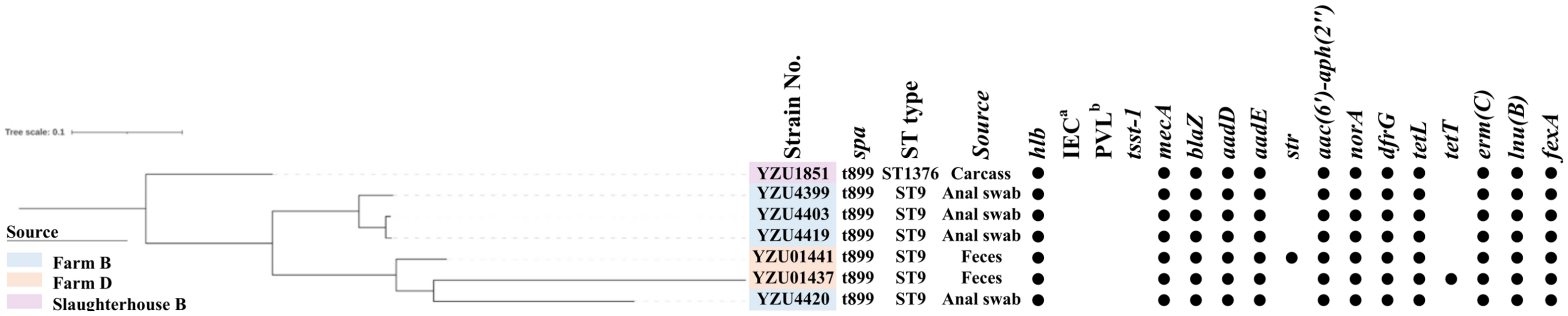
The phylogenetic tree of MRSA CC9 in terms of the core genome multi-locus sequence type (cgMLST). The filled circle (●) represents the presence of a gene. ^a^immune evasion cluster (IEC). ^b^ the Panton-Valentine leucocidin (PVL).

MLST analysis showed that MRSA isolates were grouped into two CCs, of which 56 isolates belonged to CC398 and 7 isolates belonged to CC9. MRSA CC398 isolates contained two ST types, of which 55 MRSA isolates belonged to ST398, and one isolate was a new ST type ST8505 (Figure 1). The phylogenetic analysis divided the MRSA CC398 isolates into three clusters with a predominant cluster of 44 MRSA t034/ST398 isolates, 1 t1794/ST398 isolate, and 3 ST398 isolates without *spa* type. MRSA isolates t011/ST398 and t2970/ST398 were grouped into a cluster with all isolates from farm D, while MRSA t529/ST398 were from slaughterhouse A (Figure 1). The phylogenetic analysis divided MRSA CC9 into two clusters, of which one isolate with t899/ST1378 was detected from pig carcasses in slaughterhouse B, and the other cluster with MRSA t899/ST9 isolates was from farm B and farm D (Figure 2). Virulence factors were analysed in all MRSA isolates from pig farms and slaughterhouses.

In total, 11 antibiotic resistance genes (ARGs) were detected from 56 MRSA CC398 isolates, while 18 ARGs were detected from 7 MRSA CC9 isolates. All MRSA isolates contain the *mecA* gene encoding the low-affinity penicillin-binding protein PBP 2a. Both *blaZ* encoding β-lactamase and *dfrG* for trimethoprim resistance were presented in 62 out of 63 MRSA isolates. Tetracycline-resistant genes were identified in all MRSA isolates, of which CC398 isolates harboured *tetM*, *tetT*, and *tetK*, while CC9 isolates harboured *tetT* and *tetL*. The *emr(C)* gene for the erythromycin resistance was present in 19 out of 55 MRSA CC398 isolates and all MRSA CC9 isolates, while *emr(B)* was only detected in six CC398 isolates. Aminoglycoside-resistant genes *aadD* and *aadE* were detected in CC9 isolates, while *aac(6′)-aph(2″)* was detected in all CC9 isolates and one CC398 isolate. The *norA* gene was detected in 43 out of 56 MRSA CC398 isolates and all MRSA CC9 isolates, which were associated with fluoroquinolone resistance in *S. aureus*. The *lsa(B)* gene encoding the ABC transporter for low-level clindamycin resistance was presented in 13 MRSA CC398 isolates. The macrolide-resistant gene *lnu(B)* was detected in 53 MRSA CC398 isolates and 6 MRSA CC9 isolates. Phenicol resistance genes *fexA* were detected in both CC398 and CC9 isolates, while the *cfr* gene was only detected in CC398 isolates.

To evaluate the transmission of MRSA CC398 among countries, we also conducted a core genome-based phylogenetic analysis of the 56 MRSA CC398 isolates and 67 MRSA CC398 isolates from a previous study (Price et al., 2012) (Supplementary Figure S1). The results showed that the MRSA CC398 isolates were grouped into two clusters with LA-MRSA CC398 isolates from Europe, which were genetically different from CC398 isolates from humans in China. All MRSA CC398 isolates were absent of phiSa3 carrying *chp*, *scn*, and *sak* for human-immune evasion cluster and contained an intact *hlb* gene in this study. Similar to MRSA from other countries, the *tet(M)* gene was present in all MRSA CC398 isolates from this study (Figures 1 and Supplementary Figure S1). Besides, *lukF-PV* and *lukS-PV* encoding PVL and *tsst-1* encoding toxic shock syndrome toxin-1 were also absent in all MRSA isolates (Figure 1).

## 4 Discussion

This study investigated the distribution of MRSA and MSSA from pig farms and slaughterhouses in Eastern China. In total, 11.38% of pig-associated samples were *S. aureus* positive. MRSA was detected in all four pig farms and two pig slaughterhouses, with a prevalence of 10.44% in pig farms and 15.29% in pig slaughterhouses, respectively. Other surveillance studies showed that the occurrence of MRSA in pig production was diverse in different locations in China, which is 49% in Shandong, 45% in Shanghai, 19.4% in Qinghai, and 5.17% in Henan (Li et al., 2017; Sun et al., 2019; Cui et al., 2022). The prevalence of MRSA in pig production was diverse in Western countries, with 77% in the US, 80% in Denmark, 69.7% in Belgium, and 99.5% in the Netherlands (Crombé et al., 2012; Sun et al., 2015; Dierikx et al., 2016; Schulz et al., 2019). In Japan, MRSA was detected among pigs in slaughterhouses, diseased pigs on farms, imported breeding pigs, and farm dust, with a prevalence of 5.2, 3.4, 28.8, and 0.06%, respectively (Ozawa et al., 2022). This discrepancy in MRSA in pig production suggests the diversity of MRSA distribution across different geographical locations.

WGS analysis has demonstrated that 56 out of 63 MRSA isolates in this study belonged to CC398, whereas only seven of the MRSA isolates belonged to CC9. Remarkably, MRSA CC398 was detected in all pig farms as well as in slaughterhouse A, indicating the widespread dissemination of LA-MRSA CC398 in the pig production sector of Eastern China. A similar study conducted in Qinghai province, China, detected 26 out of 67 MRSA CC398 isolates with *spa* type t011 (Cui et al., 2022), whereas this study observed the predominant LA-MRSA CC398 with *spa* type t034. In addition to the above observations, it is worth noting that the dominant clone of pig-associated LA-MRSA in China belongs to LA-MRSA CC9. Li et al. (2017) conducted a pig-associated MRSA study in four provinces in China, and all 270 MRSA isolates were classified as CC9. Furthermore, a large-scale LA-MRSA study in Shandong, China, revealed that out of 92 MRSA isolates obtained from pig samples, 91 were LA-MRSA CC9, and one isolate was LA-MRSA CC398 through WGS analysis (Sun et al., 2019). We further analysed the phylogenetic relationship of MRSA CC398 isolates in this study genome with MRSA CC398 isolates from Price et al. (2012). The results showed that all MRSA CC398 isolates from this study were closely related to the MRSA CC398 isolates from pig samples all over the world (Supplementary Figure S1). This study observed that LA-MRSA CC398 was predominated in pig farms in Eastern China for the first time, and the close phylogenetic relationship of MRSA CC398 isolates in this study indicated an emergency of global MRSA ST398 in domestic pig production.

The multidrug-resistant phenotype of MRSA isolates in this study was similar to that of MRSA strains from livestock and humans (Li et al., 2017; Cui et al., 2020; Kruger-Haker et al., 2023). All isolates were resistant to cefoxitin and tetracycline with the presence of the *mecA* gene, *tet(M)*, *tet(K)*, *tet(L)*, or *tet(T)*, which are correlated to the LA-MRSA CC398 and CC9 antimicrobial characteristics (Kock et al., 2010). Most isolates were resistant to clindamycin (95.24%) and chloramphenicol (76.19%), which were well matched with the presence of *lnu(B)* and *fexA*. Thirteen LA-MRSA CC398 isolates harboured *lnu(B)* plus *lsa(E)* encoding lincomycin and clindamycin resistance, which was detected in a previous study in the UK (Kruger-Haker et al., 2023). The presence of the *fexA* gene in LA-MRSA CC398 was reported for the first time in China, while a previous study demonstrated a low prevalence of *fexA* in CC398 in the UK (Kruger-Haker et al., 2023). A previous study demonstrated that the prevalence of *fexA* in MRSA CC9 isolates was 69.1% (Yu et al., 2021). The phenicol-lincosamide-oxazolidinone-pleuromutilin-streptogramin resistance gene *cfr* was highly prevalent in LA-MRSA CC398 isolates, and one isolate showed resistance to linezolid. Although clindamycin and linezolid are not sanctioned in pig farming, the extensive usage of other antibiotics, such as lincomycin, florfenicol, and tiamulin in food-producing animals can exert selective pressure, thereby fostering the dissemination of the *cfr* gene (Schouls et al., 2022; Kruger-Haker et al., 2023). Eight MRSA isolates contained aminoglycoside-resistant gene *aac(6′)-aph(2″),* and two isolates showed resistance to gentamicin. The emergence and proliferation of these multidrug-resistant isolates in pigs consequently impose a substantial risk upon individuals with sustained contact with livestock.

## 5 Conclusion

This study investigated the prevalence of *S. aureus* in pig production in Eastern China. MRSA has been detected in all pig farms and slaughterhouses. The high prevalence of LA-MRSA CC398 in both pig farms and slaughterhouses has provided a significant indication of the emergence and spread of LA-MRSA CC398 in the Chinese pig production industry. The phylogenetic analysis demonstrated a close relationship between LA-MRSA CC398 isolates in this study and isolates from pig samples in other countries, indicating that international trade could be a potential for LA-MRSA CC398 transmission. Further studies are required to comprehensively understand LA-MRSA CC398 in the pig industry and its potential risks to livestock and humans in the context of pork production in China.

## Data availability statement

The datasets presented in this study can be found in online repositories. The names of the repository/repositories and accession number(s) can be found in the article/Supplementary material.

## Ethics statement

The animal study was approved by the Research Ethics Committee of Yangzhou University. The study was conducted in accordance with the local legislation and institutional requirements.

## Author contributions

LZ: Data curation, Investigation, Methodology, Writing – original draft. ZJ: Data curation, Investigation, Methodology, Writing – original draft. ZW: Data curation, Formal analysis, Software, Writing – review & editing. YL: Conceptualization, Methodology, Project administration, Supervision, Writing – review & editing. XJ: Resources, Supervision, Writing – review & editing. QL: Resources, Validation, Writing – review & editing. YT: Conceptualization, Formal analysis, Funding acquisition, Supervision, Validation, Visualization, Writing – original draft, Writing – review & editing.
