# Supplementary material for: The prevalence of Staphylococcus aureus and the emergence of livestock-associated MRSA CC398 in pig production in eastern China
Source: Front Microbiol. 2023 Dec 15;14:1267885. doi: 10.3389/fmicb.2023.1267885 (PMC10755019; doi:10.3389/fmicb.2023.1267885)
Supplement: Supplementary file 1 [file Data_Sheet_1.docx]

Supplementary Material

# Supplementary Table

**Table S1.** Sampling information from pig farms and slaughterhouses

| **Sampling site** | **Location** | **Year** | **Source** | **Sample No.** | **Positive No.** | **Prevalence** | **MSSA** | **MRSA** |
| --- | --- | --- | --- | --- | --- | --- | --- | --- |
| **Farm A** | Danyang, Jiangsu | 2021 | Feces | 105 | 1 | 0.95% | 0 | 1 |
|  |  |  | Anal swabs | 371 | 6 | 1.62% | 0 | 6 |
|  |  |  | Nasal swabs | 105 | 4 | 3.81% | 0 | 4 |
|  |  |  | Gloves | 98 | 2 | 2.04% | 0 | 2 |
| **Farm B** | Danyang, Jiangsu | 2021 | Anal swabs | 170 | 22 | 12.94% | 0 | 22 |
|  |  |  | Feces | 15 | 4 | 26.67% | 0 | 4 |
|  |  |  | Gloves | 15 | 3 | 20.00% | 0 | 3 |
| **Farm C** | Danyang, Jiangsu | 2021 | Feces | 60 | 11 | 18.33% | 0 | 11 |
| **Farm D** | Shanghai | 2018 | Feces | 124 | 58 | 46.77% | 50 | 8 |
| **Slaugtherhouse A** | Huai'an, Jiangsu | 2019 | Depilation | 20 | 1 | 5.00% | 1 | 0 |
|  |  |  | Splitting | 20 | 2 | 10.00% | 2 | 0 |
|  |  |  | Dressing | 20 | 2 | 10.00% | 2 | 0 |
|  |  |  | Chilling | 20 | 1 | 5.00% | 1 | 0 |
|  |  |  | Before slaughtering | 20 | 2 | 10.00% | 1 | 1 |
|  |  |  | Washing | 20 | 0 | 0.00% | 0 | 0 |
|  |  |  | Evisceriation | 10 | 0 | 0.00% | 0 | 0 |
|  |  |  | Floor | 5 | 0 | 0.00% | 0 | 0 |
|  |  |  | Knives for bleeding | 10 | 0 | 0.00% | 0 | 0 |
|  |  |  | Knives for scalding | 10 | 0 | 0.00% | 0 | 0 |
|  |  |  | Knives for splitting | 10 | 1 | 10.00% | 1 | 0 |
| **Slaugtherhouse B** | Jiangdu, Jiangsu | 2019 | Bleeding | 10 | 1 | 10.00% | 1 | 0 |
|  |  |  | Depilation | 10 | 1 | 10.00% | 0 | 1 |
|  |  |  | Splitting | 10 | 7 | 70.00% | 7 | 0 |
|  |  |  | Dressing | 6 | 6 | 100.00% | 6 | 0 |
|  |  |  | Chilling | 10 | 6 | 60.00% | 6 | 0 |
|  |  |  | Before slaughtering | 10 | 0 | 0.00% | 0 | 0 |
|  |  |  | Scalding | 10 | 1 | 10.00% | 1 | 0 |
|  |  |  | Evisceriation | 1 | 0 | 0.00% | 0 | 0 |
|  |  |  | Floor | 3 | 0 | 0.00% | 0 | 0 |
|  |  |  | Knives for bleeding | 5 | 1 | 20.00% | 1 | 0 |
|  |  |  | Knives for scalding | 5 | 4 | 80.00% | 4 | 0 |
|  |  |  | Knives for dressing | 5 | 2 | 40.00% | 2 | 0 |
|  |  |  | Knives for splitting | 5 | 1 | 20.00% | 1 | 0 |
| **Total** | **/** | **/** | **/** | **1318** | **150** | **11.38%** | **87** | **63** |

**Table S2.** Information of MRSA isolates from pig farms and slaughterhouses.

| **Strain No.** | **STs** | ***spa* Type** | **Source** | **Sampling site** | **Sampling date** | **Location** |
| --- | --- | --- | --- | --- | --- | --- |
| YZU4613 | 398 | t034 | Feces | Farm A | 2021 | Danyang, Jiangsu |
| YZU4614 | 8505 | t034 | Anal swab | Farm A | 2021 | Danyang, Jiangsu |
| YZU4615 | 398 | t034 | Anal swab | Farm A | 2021 | Danyang, Jiangsu |
| YZU4616 | 398 | t034 | Anal swab | Farm A | 2021 | Danyang, Jiangsu |
| YZU4617 | 398 | t034 | Anal swab | Farm A | 2021 | Danyang, Jiangsu |
| YZU4618 | 398 | t034 | Anal swab | Farm A | 2021 | Danyang, Jiangsu |
| YZU4619 | 398 | t034 | Anal swab | Farm A | 2021 | Danyang, Jiangsu |
| YZU4623 | 398 | t034 | Nasal swab | Farm A | 2021 | Danyang, Jiangsu |
| YZU4622 | 398 | t034 | Nasal swab | Farm A | 2021 | Danyang, Jiangsu |
| YZU4624 | 398 | t034 | Nasal swab | Farm A | 2021 | Danyang, Jiangsu |
| YZU4625 | 398 | t034 | Nasal swab | Farm A | 2021 | Danyang, Jiangsu |
| YZU4620 | 398 | NA | Glove | Farm A | 2021 | Danyang, Jiangsu |
| YZU4621 | 398 | NA | Glove | Farm A | 2021 | Danyang, Jiangsu |
| YZU4404 | 398 | t034 | Feces | Farm B | 2021 | Danyang, Jiangsu |
| YZU4405 | 398 | t034 | Feces | Farm B | 2021 | Danyang, Jiangsu |
| YZU4406 | 398 | t034 | Feces | Farm B | 2021 | Danyang, Jiangsu |
| YZU4407 | 398 | t034 | Feces | Farm B | 2021 | Danyang, Jiangsu |
| YZU4391 | 398 | t034 | Anal swab | Farm B | 2021 | Danyang, Jiangsu |
| YZU4393 | 398 | t034 | Anal swab | Farm B | 2021 | Danyang, Jiangsu |
| YZU4394 | 398 | t034 | Anal swab | Farm B | 2021 | Danyang, Jiangsu |
| YZU4395 | 398 | t034 | Anal swab | Farm B | 2021 | Danyang, Jiangsu |
| YZU4414 | 398 | t034 | Anal swab | Farm B | 2021 | Danyang, Jiangsu |
| YZU4397 | 398 | t034 | Anal swab | Farm B | 2021 | Danyang, Jiangsu |
| YZU4411 | 398 | t034 | Anal swab | Farm B | 2021 | Danyang, Jiangsu |
| YZU4412 | 398 | t034 | Anal swab | Farm B | 2021 | Danyang, Jiangsu |
| YZU4413 | 398 | t034 | Anal swab | Farm B | 2021 | Danyang, Jiangsu |
| YZU4415 | 398 | t034 | Anal swab | Farm B | 2021 | Danyang, Jiangsu |
| YZU4416 | 398 | t034 | Anal swab | Farm B | 2021 | Danyang, Jiangsu |
| YZU4417 | 398 | t034 | Anal swab | Farm B | 2021 | Danyang, Jiangsu |
| YZU4418 | 398 | t034 | Anal swab | Farm B | 2021 | Danyang, Jiangsu |
| YZU4419 | 9 | t899 | Anal swab | Farm B | 2021 | Danyang, Jiangsu |
| YZU4420 | 9 | t899 | Anal swab | Farm B | 2021 | Danyang, Jiangsu |
| YZU4421 | 398 | t034 | Anal swab | Farm B | 2021 | Danyang, Jiangsu |
| YZU4398 | 398 | t034 | Anal swab | Farm B | 2021 | Danyang, Jiangsu |
| YZU4399 | 9 | t899 | Anal swab | Farm B | 2021 | Danyang, Jiangsu |
| YZU4400 | 398 | t034 | Anal swab | Farm B | 2021 | Danyang, Jiangsu |
| YZU4401 | 398 | t034 | Anal swab | Farm B | 2021 | Danyang, Jiangsu |
| YZU4402 | 398 | t034 | Anal swab | Farm B | 2021 | Danyang, Jiangsu |
| YZU4403 | 9 | t899 | Anal swab | Farm B | 2021 | Danyang, Jiangsu |
| YZU4408 | 398 | t034 | Glove | Farm B | 2021 | Danyang, Jiangsu |
| YZU4409 | 398 | t1793 | Glove | Farm B | 2021 | Danyang, Jiangsu |
| YZU4410 | 398 | t034 | Glove | Farm B | 2021 | Danyang, Jiangsu |
| YZU4382 | 398 | NA | Feces | Farm C | 2021 | Danyang, Jiangsu |
| YZU4387 | 398 | t034 | Feces | Farm C | 2021 | Danyang, Jiangsu |
| YZU4383 | 398 | t034 | Feces | Farm C | 2021 | Danyang, Jiangsu |
| YZU4384 | 398 | t034 | Feces | Farm C | 2021 | Danyang, Jiangsu |
| YZU4385 | 398 | t034 | Feces | Farm C | 2021 | Danyang, Jiangsu |
| YZU4386 | 398 | t034 | Feces | Farm C | 2021 | Danyang, Jiangsu |
| YZU4380 | 398 | t034 | Feces | Farm C | 2021 | Danyang, Jiangsu |
| YZU4381 | 398 | t034 | Feces | Farm C | 2021 | Danyang, Jiangsu |
| YZU4388 | 398 | t034 | Feces | Farm C | 2021 | Danyang, Jiangsu |
| YZU4389 | 398 | t034 | Feces | Farm C | 2021 | Danyang, Jiangsu |
| YZU4390 | 398 | t034 | Feces | Farm C | 2021 | Danyang, Jiangsu |
| YZU01432 | 398 | t011 | Feces | Farm D | 2018 | Shanghai |
| YZU01435 | 398 | t011 | Feces | Farm D | 2018 | Shanghai |
| YZU01437 | 9 | t899 | Feces | Farm D | 2018 | Shanghai |
| YZU01439 | 398 | t011 | Feces | Farm D | 2018 | Shanghai |
| YZU01440 | 398 | t011 | Feces | Farm D | 2018 | Shanghai |
| YZU01441 | 9 | t899 | Feces | Farm D | 2018 | Shanghai |
| YZU01443 | 398 | t011 | Feces | Farm D | 2018 | Shanghai |
| YZU01445 | 398 | t2970 | Feces | Farm D | 2018 | Shanghai |
| YZU1845 | 398 | t529 | After slaughtering | Slaughterhouse A | 2019 | Jiangdu, Jiangsu |
| YZU1851 | 1376 | t899 | Depilation | Slaughterhouse B | 2019 | Huai‘an, Jiangsu |

**Table S3.**  Information of MSSA isolates from pig farms and slaughterhouses.

| **No.** | **Strain No.** | ***spa* type** | **Source** | **Sampling site** | **Sampling date** | **Location** |
| --- | --- | --- | --- | --- | --- | --- |
| 1 | LQSA19301 | t571 | After slaughtering | Slaughterhouse A | 2019 | Jiangdu, Jiangsu |
| 2 | LQSA19295 | t571 | Depilation | Slaughterhouse A | 2019 | Jiangdu, Jiangsu |
| 3 | LQSA19296 | t571 | Splitting | Slaughterhouse A | 2019 | Jiangdu, Jiangsu |
| 4 | LQSA19297 | t571 | Splitting | Slaughterhouse A | 2019 | Jiangdu, Jiangsu |
| 5 | LQSA19303 | t571 | Knives for splitting | Slaughterhouse A | 2019 | Jiangdu, Jiangsu |
| 6 | LQSA19298 | t899 | Dressing | Slaughterhouse A | 2019 | Jiangdu, Jiangsu |
| 7 | LQSA19299 | t571 | Dressing | Slaughterhouse A | 2019 | Jiangdu, Jiangsu |
| 8 | LQSA19302 | t571 | Chilling | Slaughterhouse A | 2019 | Jiangdu, Jiangsu |
| 9 | LQSA19177 | t286 | Bleeding | Slaughterhouse B | 2019 | Huai‘an, Jiangsu |
| 10 | LQSA19193 | t286 | Knives for bleeding | Slaughterhouse B | 2019 | Huai‘an, Jiangsu |
| 11 | LQSA19196 | t286 | Scalding | Slaughterhouse B | 2019 | Huai‘an, Jiangsu |
| 12 | LQSA19185 | t286 | Knives for scalding | Slaughterhouse B | 2019 | Huai‘an, Jiangsu |
| 13 | LQSA19192 | t011 | Knives for scalding | Slaughterhouse B | 2019 | Huai‘an, Jiangsu |
| 14 | LQSA19197 | t286 | Knives for scalding | Slaughterhouse B | 2019 | Huai‘an, Jiangsu |
| 15 | LQSA19201 | t286 | Knives for scalding | Slaughterhouse B | 2019 | Huai‘an, Jiangsu |
| 16 | LQSA19175 | t286 | Splitting | Slaughterhouse B | 2019 | Huai‘an, Jiangsu |
| 17 | LQSA19176 | t286 | Splitting | Slaughterhouse B | 2019 | Huai‘an, Jiangsu |
| 18 | LQSA19180 | t286 | Splitting | Slaughterhouse B | 2019 | Huai‘an, Jiangsu |
| 19 | LQSA19181 | t286 | Splitting | Slaughterhouse B | 2019 | Huai‘an, Jiangsu |
| 20 | LQSA19182 | t286 | Splitting | Slaughterhouse B | 2019 | Huai‘an, Jiangsu |
| 21 | LQSA19184 | t286 | Splitting | Slaughterhouse B | 2019 | Huai‘an, Jiangsu |
| 22 | LQSA19189 | t286 | Splitting | Slaughterhouse B | 2019 | Huai‘an, Jiangsu |
| 23 | LQSA19203 | t237 | Knives for splitting | Slaughterhouse B | 2019 | Huai‘an, Jiangsu |
| 24 | LQSA19178 | t1775 | Dressing | Slaughterhouse B | 2019 | Huai‘an, Jiangsu |
| 25 | LQSA19179 | t286 | Dressing | Slaughterhouse B | 2019 | Huai‘an, Jiangsu |
| 26 | LQSA19183 | t286 | Dressing | Slaughterhouse B | 2019 | Huai‘an, Jiangsu |
| 27 | LQSA19187 | t286 | Dressing | Slaughterhouse B | 2019 | Huai‘an, Jiangsu |
| 28 | LQSA19194 | t286 | Dressing | Slaughterhouse B | 2019 | Huai‘an, Jiangsu |
| 29 | LQSA19199 | t286 | Dressing | Slaughterhouse B | 2019 | Huai‘an, Jiangsu |
| 30 | LQSA19190 | t286 | Knives for dressing | Slaughterhouse B | 2019 | Huai‘an, Jiangsu |
| 31 | LQSA19191 | t286 | Knives for dressing | Slaughterhouse B | 2019 | Huai‘an, Jiangsu |
| 32 | LQSA19186 | t286 | Chilling | Slaughterhouse B | 2019 | Huai‘an, Jiangsu |
| 33 | LQSA19188 | t286 | Chilling | Slaughterhouse B | 2019 | Huai‘an, Jiangsu |
| 34 | LQSA19195 | t286 | Chilling | Slaughterhouse B | 2019 | Huai‘an, Jiangsu |
| 35 | LQSA19198 | t286 | Chilling | Slaughterhouse B | 2019 | Huai‘an, Jiangsu |
| 36 | LQSA19200 | t286 | Chilling | Slaughterhouse B | 2019 | Huai‘an, Jiangsu |
| 37 | LQSA19202 | t286 | Chilling | Slaughterhouse B | 2019 | Huai‘an, Jiangsu |
| 38 | LQSA19001 | t1775 | Feces | Farm D | 2018 | Shanghai |
| 39 | LQSA19002 | t899 | Feces | Farm D | 2018 | Shanghai |
| 40 | LQSA19003 | t571 | Feces | Farm D | 2018 | Shanghai |
| 41 | LQSA19004 | t899 | Feces | Farm D | 2018 | Shanghai |
| 42 | LQSA19005 | t571 | Feces | Farm D | 2018 | Shanghai |
| 43 | LQSA19006 | t571 | Feces | Farm D | 2018 | Shanghai |
| 44 | LQSA19007 | t571 | Feces | Farm D | 2018 | Shanghai |
| 45 | LQSA19008 | t571 | Feces | Farm D | 2018 | Shanghai |
| 46 | LQSA19009 | t899 | Feces | Farm D | 2018 | Shanghai |
| 47 | LQSA19010 | t899 | Feces | Farm D | 2018 | Shanghai |
| 48 | LQSA19011 | t571 | Feces | Farm D | 2018 | Shanghai |
| 49 | LQSA19012 | t899 | Feces | Farm D | 2018 | Shanghai |
| 50 | LQSA19013 | t571 | Feces | Farm D | 2018 | Shanghai |
| 51 | LQSA19014 | t571 | Feces | Farm D | 2018 | Shanghai |
| 52 | LQSA19015 | t571 | Feces | Farm D | 2018 | Shanghai |
| 53 | LQSA19016 | t571 | Feces | Farm D | 2018 | Shanghai |
| 54 | LQSA19017 | t571 | Feces | Farm D | 2018 | Shanghai |
| 55 | LQSA19018 | t571 | Feces | Farm D | 2018 | Shanghai |
| 56 | LQSA19019 | t571 | Feces | Farm D | 2018 | Shanghai |
| 57 | LQSA19020 | NA | Feces | Farm D | 2018 | Shanghai |
| 58 | LQSA19021 | NA | Feces | Farm D | 2018 | Shanghai |
| 59 | LQSA19025 | t571 | Feces | Farm D | 2018 | Shanghai |
| 60 | LQSA19026 | t571 | Feces | Farm D | 2018 | Shanghai |
| 61 | LQSA19032 | t899 | Feces | Farm D | 2018 | Shanghai |
| 62 | LQSA19040 | t1456 | Feces | Farm D | 2018 | Shanghai |
| 63 | LQSA19042 | t011 | Feces | Farm D | 2018 | Shanghai |
| 64 | LQSA19043 | t1456 | Feces | Farm D | 2018 | Shanghai |
| 65 | LQSA19044 | t286 | Feces | Farm D | 2018 | Shanghai |
| 66 | LQSA19045 | t1775 | Feces | Farm D | 2018 | Shanghai |
| 67 | LQSA19046 | t1776 | Feces | Farm D | 2018 | Shanghai |
| 68 | LQSA19047 | t1775 | Feces | Farm D | 2018 | Shanghai |
| 69 | LQSA19048 | t1775 | Feces | Farm D | 2018 | Shanghai |
| 70 | LQSA19049 | t1456 | Feces | Farm D | 2018 | Shanghai |
| 71 | LQSA19050 | t1456 | Feces | Farm D | 2018 | Shanghai |
| 72 | LQSA19051 | t1456 | Feces | Farm D | 2018 | Shanghai |
| 73 | LQSA19052 | t011 | Feces | Farm D | 2018 | Shanghai |
| 74 | LQSA19053 | t899 | Feces | Farm D | 2018 | Shanghai |
| 75 | LQSA19054 | t899 | Feces | Farm D | 2018 | Shanghai |
| 76 | LQSA19055 | t899 | Feces | Farm D | 2018 | Shanghai |
| 77 | LQSA19056 | t899 | Feces | Farm D | 2018 | Shanghai |
| 78 | LQSA19057 | t899 | Feces | Farm D | 2018 | Shanghai |
| 79 | LQSA19058 | t899 | Feces | Farm D | 2018 | Shanghai |
| 80 | LQSA19059 | t286 | Feces | Farm D | 2018 | Shanghai |
| 81 | LQSA19060 | t1456 | Feces | Farm D | 2018 | Shanghai |
| 82 | LQSA19061 | t899 | Feces | Farm D | 2018 | Shanghai |
| 83 | LQSA19062 | t899 | Feces | Farm D | 2018 | Shanghai |
| 84 | LQSA19063 | t1775 | Feces | Farm D | 2018 | Shanghai |
| 85 | LQSA19064 | t1775 | Feces | Farm D | 2018 | Shanghai |
| 86 | LQSA19065 | t1775 | Feces | Farm D | 2018 | Shanghai |
| 87 | LQSA19066 | t571 | Feces | Farm D | 2018 | Shanghai |

**Table S4**. The multidrug resistant patterns of MRSA isolates from pig farm and slaughterhouse

| **Strain No.** | **Multidrug resistant pattern** | **FOX** | **C** | **CIP** | **DA** | **E** | **CN** | **LZD** | **P** | **TE** | **SXT** |
| --- | --- | --- | --- | --- | --- | --- | --- | --- | --- | --- | --- |
| YZU1845 | FOX-DA-P-TE | FOX |  |  | DA |  |  |  | P | TE |  |
| YZU01445 | FOX-DA-E-P-TE | FOX |  |  | DA | E |  |  | P | TE |  |
| YZU01439 | FOX-DA-E-P-TE | FOX |  |  | DA | E |  |  | P | TE |  |
| YZU01443 | FOX-DA-E-P-TE | FOX |  |  | DA | E |  |  | P | TE |  |
| YZU01440 | FOX-CIP-E-P-TE | FOX |  | CIP |  | E |  |  | P | TE |  |
| YZU01435 | FOX-CIP-DA-E-P-TE | FOX |  | CIP | DA | E |  |  | P | TE |  |
| YZU01432 | FOX-C-DA-E-P-TE | FOX | C |  | DA | E |  |  | P | TE |  |
| YZU4388 | FOX-C-DA-E-P-TE | FOX | C |  | DA | E |  |  | P | TE |  |
| YZU4385 | FOX-C-DA-E-LZD-P-TE | FOX | C |  | DA | E |  | LZD | P | TE |  |
| YZU4386 | FOX-C-DA-E-P-TE | FOX | C |  | DA | E |  |  | P | TE |  |
| YZU4389 | FOX-C-DA-E-P-TE | FOX | C |  | DA | E |  |  | P | TE |  |
| YZU4384 | FOX-C-DA-E-P-TE | FOX | C |  | DA | E |  |  | P | TE |  |
| YZU4390 | FOX-C-E-P-TE | FOX | C |  |  | E |  |  | P | TE |  |
| YZU4614 | FOX-C-DA-P-TE | FOX | C |  | DA |  |  |  | P | TE |  |
| YZU4618 | FOX-C-DA-P-TE | FOX | C |  | DA |  |  |  | P | TE |  |
| YZU4619 | FOX-C-DA-E-P-TE | FOX | C |  | DA | E |  |  | P | TE |  |
| YZU4382 | FOX-C-DA-E-P-TE | FOX | C |  | DA | E |  |  | P | TE |  |
| YZU4621 | FOX-C-DA-E-P-TE | FOX | C |  | DA | E |  |  | P | TE |  |
| YZU4620 | FOX-C-DA-E-P-TE | FOX | C |  | DA | E |  |  | P | TE |  |
| YZU4387 | FOX-C-DA-E-P-TE | FOX | C |  | DA | E |  |  | P | TE |  |
| YZU4409 | FOX-DA-P-TE | FOX |  |  | DA |  |  |  | P | TE |  |
| YZU4405 | FOX-DA-P-TE | FOX |  |  | DA |  |  |  | P | TE |  |
| YZU4395 | FOX-C-DA-P-TE | FOX | C |  | DA |  |  |  | P | TE |  |
| YZU4411 | FOX-C-DA-P-TE | FOX | C |  | DA |  |  |  | P | TE |  |
| YZU4417 | FOX-C-DA-P-TE | FOX | C |  | DA |  |  |  | P | TE |  |
| YZU4415 | FOX-C-DA-P-TE | FOX | C |  | DA |  |  |  | P | TE |  |
| YZU4393 | FOX-DA-P-TE | FOX |  |  | DA |  |  |  | P | TE |  |
| YZU4406 | FOX-DA-P-TE | FOX |  |  | DA |  |  |  | P | TE |  |
| YZU4413 | FOX-C-DA-P-TE | FOX | C |  | DA |  |  |  | P | TE |  |
| YZU4391 | FOX-C-DA-E-P-TE | FOX | C |  | DA | E |  |  | P | TE |  |
| YZU4408 | FOX-C-DA-P-TE | FOX | C |  | DA |  |  |  | P | TE |  |
| YZU4414 | FOX-C-DA-P-TE | FOX | C |  | DA |  |  |  | P | TE |  |
| YZU4404 | FOX-C-DA-P-TE | FOX | C |  | DA |  |  |  | P | TE |  |
| YZU4616 | FOX-DA-E-P-TE | FOX |  |  | DA | E |  |  | P | TE |  |
| YZU4617 | FOX-DA-E-P-TE | FOX |  |  | DA | E |  |  | P | TE |  |
| YZU4615 | FOX-DA-E-P-TE | FOX |  |  | DA | E |  |  | P | TE |  |
| YZU4613 | FOX-DA-P-TE | FOX |  |  | DA |  |  |  | P | TE |  |
| YZU4623 | FOX-C-DA-P-TE | FOX | C |  | DA |  |  |  | P | TE |  |
| YZU4625 | FOX-C-DA-P-TE | FOX | C |  | DA |  |  |  | P | TE |  |
| YZU4624 | FOX-C-DA-P-TE | FOX | C |  | DA |  |  |  | P | TE |  |
| YZU4622 | FOX-C-DA-P-TE | FOX | C |  | DA |  |  |  | P | TE |  |
| YZU4381 | FOX-C-DA-E-P-TE | FOX | C |  | DA | E |  |  | P | TE |  |
| YZU4380 | FOX-C-DA-E-P-TE | FOX | C |  | DA | E |  |  | P | TE |  |
| YZU4416 | FOX-C-DA-E-P-TE | FOX | C |  | DA | E |  |  | P | TE |  |
| YZU4407 | FOX-C-DA-P-TE | FOX | C |  | DA |  |  |  | P | TE |  |
| YZU4412 | FOX-C-DA-P-TE | FOX | C |  | DA |  |  |  | P | TE |  |
| YZU4421 | FOX-C-DA-P-TE | FOX | C |  | DA |  |  |  | P | TE |  |
| YZU4410 | FOX-C-DA-P-TE | FOX | C |  | DA |  |  |  | P | TE |  |
| YZU4398 | FOX-C-DA-P-TE | FOX | C |  | DA |  |  |  | P | TE |  |
| YZU4397 | FOX-C-DA-P-TE | FOX | C |  | DA |  |  |  | P | TE |  |
| YZU4383 | FOX-C-E-P-TE | FOX | C |  |  | E |  |  | P | TE |  |
| YZU4402 | FOX-C-DA-P-TE | FOX | C |  | DA |  |  |  | P | TE |  |
| YZU4400 | FOX-C-DA-P-TE | FOX | C |  | DA |  |  |  | P | TE |  |
| YZU4394 | FOX-C-DA-P-TE | FOX | C |  | DA |  |  |  | P | TE |  |
| YZU4418 | FOX-C-DA-P-TE | FOX | C |  | DA |  |  |  | P | TE |  |
| YZU4401 | FOX-C-DA-P-TE | FOX | C |  | DA |  |  |  | P | TE |  |
| YZU1851 | FOX-C-CIP-DA-E-P-TE | FOX | C | CIP | DA | E |  |  | P | TE |  |
| YZU4399 | FOX-C-DA-P-TE | FOX | C |  | DA |  |  |  | P | TE |  |
| YZU4403 | FOX-CIP-DA-E-CN-P-TE-SXT | FOX |  | CIP | DA | E | CN |  | P | TE | SXT |
| YZU4419 | FOX-C-DA-E-P-TE | FOX | C |  | DA | E |  |  | P | TE |  |
| YZU01441 | FOX-C-DA-E-P-TE | FOX | C |  | DA | E |  |  | P | TE |  |
| YZU01437 | FOX-C-DA-E-CN-P-TE | FOX | C |  | DA | E | CN |  | P | TE |  |
| YZU4420 | FOX-C-DA-E-P-TE | FOX | C |  | DA | E |  |  | P | TE |  |
| **Total** | **20** | **63 (100%)** | **48 (76.19%)** | **4 (6.35%)** | **60 (95.24%)** | **31 (49.21%)** | **2 (3.17%)** | **9 (14.29%)** | **1 (1.59%)** | **63 (100%)** | **1 (1.59%)** |

FOX: Cefoxitin (30 µg); C: Chloramphenicol (30 µg); CIP: Ciprofloxacin (5 µg); DA: Clindamycin (2 µg); E: Erythromycin (15 µg); CN: Gentamicin (10 µg); K: Kanamycin (30 µg); LZD: Linezolid (10 µg); TE: Tetracycline (30 µg); SXT: Trimethoprim/Sulfamethoxazole (1:19, 25 µg)

# Supplementary Figure


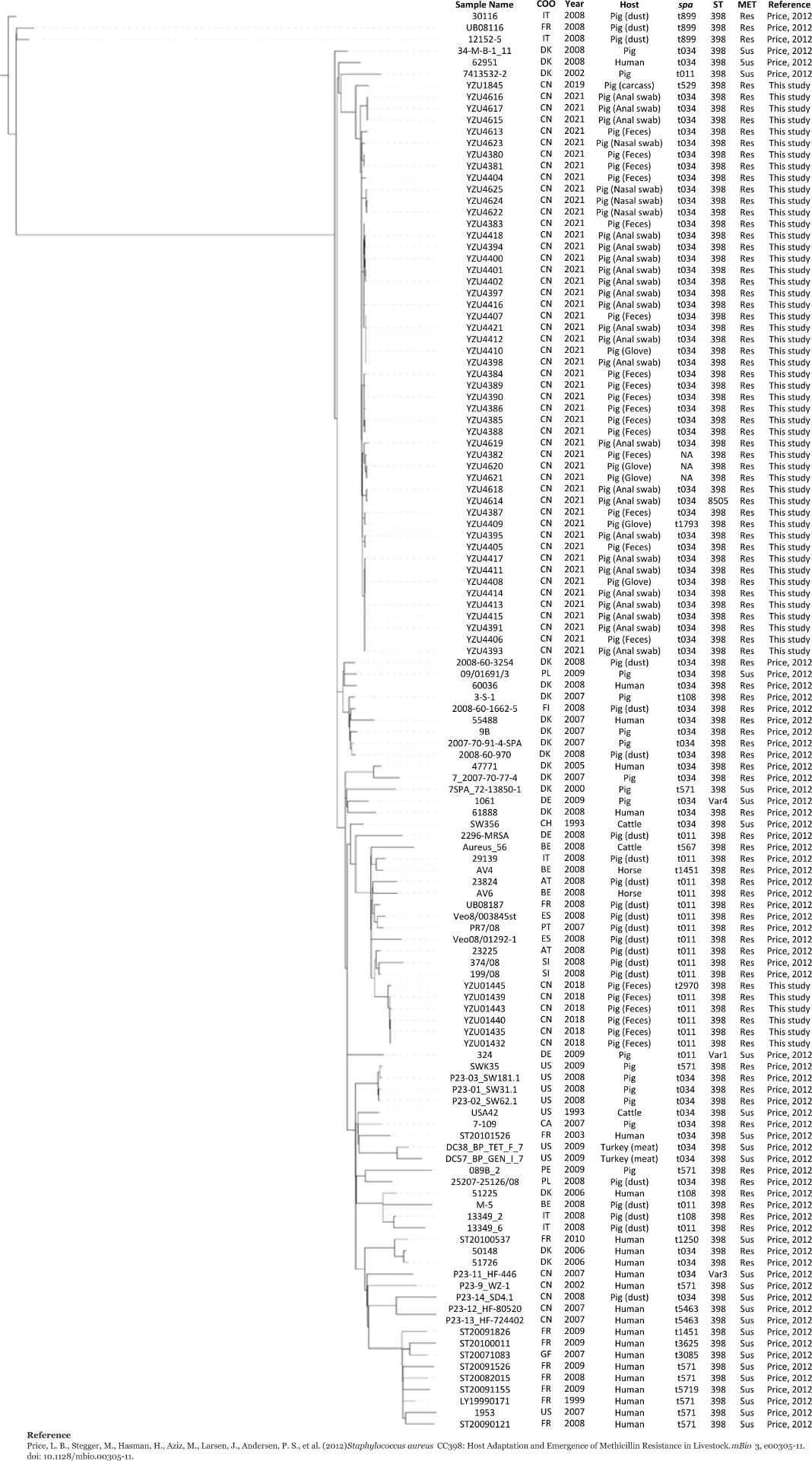


**Figure S1.** The phylogenetic tree of LA-MRSA CC398 in terms of the core genome. Strains from other countries were from a previous study by Price *et al.*(2012). . COO, country of origin; AT, Austria; BE, Belgium; CA, Canada; CH, Switzerland; CN, China; DE, Germany; DK, Denmark; ES, Spain; FI, Finland; FR, France; GF, French Guiana; HU, Hungary; IT, Italy; NL, The Netherlands; PE, Peru; PL, Poland; PT, Portugal; SI, Slovenia; US, United States; Res, resistant; Sus, susceptible.
